# Biological characteristics of human menstrual blood‐derived endometrial stem cells

**DOI:** 10.1111/jcmm.13437

**Published:** 2017-12-26

**Authors:** Yanli Liu, Rongcheng Niu, Fen Yang, Yan Yan, Shengying Liang, Yuliang Sun, Ping Shen, Juntang Lin

**Affiliations:** ^1^ Henan Key Laboratory of Medical Tissue Regeneration Stem Cells & Biotherapy Engineering Research Center of Henan College of Life Science and Technology Xinxiang Medical University Xinxiang China; ^2^ Deutsches Rheuma‐Forschungszentrum, a Leibniz Institute Berlin Germany; ^3^ Institute of Anatomy I Jena University Hospital University of Jena School of Medicine Jena Germany

**Keywords:** menstrual blood‐derived endometrial stem cells, biological characteristics, stem cell‐based therapy, paracrine effect, immunomodulation

## Abstract

Successful isolation of human endometrial stem cells from menstrual blood, namely menstrual blood‐derived endometrial stem cells (MenSCs), has provided enticing alternative seed cells for stem cell‐based therapy. MenSCs are enriched in the self‐regenerative tissue, endometrium, which shed along the periodic menstrual blood and thus their acquisition involves no physical invasiveness. However, the impact of the storage duration of menstrual blood prior to stem cell isolation, the age of the donor, the number of passages on the self‐renewing of MenSCs, the paracrine production of biological factors in MenSCs and expression of adhesion molecules on MenSCs remain elusive. In this study, we confirmed that MenSCs reside in shedding endometrium, and documented that up to 3 days of storage at 4°C has little impact on MenSCs, while the age of the donor and the number of passages are negatively associated with proliferation capacity of MenSCs. Moreover, we found that MenSCs were actually immune‐privileged and projected no risk of tumour formation. Also, we documented a lung‐ and liver‐dominated, spleen‐ and kidney‐involved organic distribution profile of MenSC 3 days after intravenous transfer into mice. At last, we suggested that MenSCs may have potentially therapeutic effects on diseases through paracrine effect and immunomodulation.

## Introduction

During the past decade, the laboratory data and clinical trials have established the knowledge that adult stem cell (ASC)‐based therapy could improve a myriad of diseases due to their intrinsic potential of regeneration and immunoregulation 1, 2, 3. The origin of ASC is known to play an important role in sustaining their distinct functional properties, which most likely contribute to their particular therapeutic effects 3, 4. Clinical trials up to date have often used cells derived from tissues such as bone marrow, adipose and umbilical cord. However, the critical problems such as invasive sample collection and rare sources limit the extensive application of these types of stem cells 5, 6, 7, 8.

As a newly identified type of ASC, human menstrual blood‐derived endometrial stem cells (MenSCs) were first reported in 2007 and show advantages with regard to non‐invasive collection procedure, high proliferation rate, multipotency, low immunogenicity and risk of karyotypic abnormalities, comparing to the other types of stem cell 9, 10, 11. Besides following the classic definition of ASC, which is positive for CD29, CD44, CD73, CD90 and CD105 and negative for haematopoietic cell surface markers, including CD34, CD45 and CD133, MenSCs also express embryonic stem cell (ESC) markers alike OCT‐4, SOX2 and SSEA‐4 11, 12, 13. Meanwhile, the therapeutic potentials of MenSCs have also been evaluated in multiple sclerosis, Duchenne muscular dystrophy, congestive heart failure and other diseases. As expected, the therapeutic effects are promising, and no adverse effects were observed during the subsequent follow‐ups 14, 15, 16, 17, 18.

The aim of this study was to firstly confirm the localization of MenSCs; subsequently address the influence of storage duration of menstrual blood preceding stem cell isolation, the age of the female donors and the number of passages on the quality of MenSCs; thirdly investigate the immunogenicity, tumorigenicity and organic distribution of MenSCs upon adoptive transfer; and finally determine the potential paracrine effect of MenSCs and the expression of adhesion molecules on MenSCs.

## Materials and methods

### Cells and animals

All the MenSCs used in this study were harvested with the informed consent of the donors. PC12 cells were kept in our laboratory. This study was approved by Ethics Committee of the Xinxiang Medical University, and experimental procedures for menstrual blood samples and MenSC were carried out in accordance with the approved guidelines. We received informed consents from all of the donors, and all the donors provided consent for the use of their menstrual blood samples in scientific research.

Six‐ to eight‐week‐old BALB/c mice (20–25 g) and nude mice‐BALB/c were purchased from Vital River Laboratories (Beijing, China). Handling of mice and experimental procedures were carried out in accordance with the guidelines of Animal Care Committee of Xinxiang Medical University. The mice were bred and housed in a specific pathogen‐free condition on a 12 hrs light–dark cycle.

### Isolation and culture of MenSCs

The menstrual blood samples were collected from healthy female donors by menstrual cup during the first few days of menses, and the samples were mixed with equal volume of phosphate‐buffered saline (PBS) containing 0.25 mg/ml amphotericin B, 100 U/ml penicillin, 100 mg/ml streptomycin and 2 mM ethylenediaminetetraacetic acid (EDTA). Shortly thereafter, the part of the supernatant of the samples was used to check the contamination of bacteria, and menstrual blood samples were delivered into laboratory and subject to standard Ficoll procedures within 72 hrs. After centrifugation, the karyocytes and the deciduous endometrium suspending in buffy coat were transferred into new tube, then washed in PBS for twice, generally suspended in growth medium [high‐glucose DMEM medium (Hyclone, USA) supplemented with 10% FBS (Gibco, USA), 100 U/ml penicillin and 100 mg/ml streptomycin] and seeded into 25 cm^2^ plastic cell culture flasks at 37°C with 5% humidified CO_2_. After 2 days of incubation, the non‐adherent cells were washed away leaving behind the adherent cells that were growing as fibroblastic cells in clusters and the medium was replaced every 3 days. When the cells reaching 80–90% confluence (P0), the cells were detached by 0.25% trypsin/1 mM EDTA and subcultured to new flasks by the ratio of 1:3.

In addition, for confirming the localization of MenSCs, the procedures including natural sedimentation and stainless steel mesh filtration were used. After density gradient centrifugation, the karyocytes and the visible deciduous endometrium suspending in buffy coat were firstly separated by 2 min. standing; subsequently, the cell suspensions without visible sediments were filtered through a 100‐μm stainless steel mesh; finally, these three kinds of samples (visible sediments, suspensions without visible sediments and filtered suspensions) were seeded into 6‐well plate and cultured as described above.

### Proliferation capacity

To assess the proliferative capacity of different passages of MenSCs (P3, P9 and P18) among 20 ± 2‐year, 30 ± 2‐year and 40 ± 2‐year groups (*n* = 6 for each group), the cells were suspended in growth medium and respectively seeded at the density of 1.25 × 10^4^ cells/ml, 2.5 × 10^4^ cells/ml and 5 × 10^4^ cells/ml into 96‐well plates. After incubation at 37°C with 5% humidified CO_2_ for 1, 3, 5, 7 and 9 days, respectively, proliferative response was determined by MTT assay, and the absorbances were analysed at 490 nm.

### Immunophenotyping analysis

MenSCs harvested from different passages were used for immunophenotyping analysis. Mouse anti‐human monoclonal antibodies: FITC‐conjugated CD29, CD73, CD90, HLA‐ABC, HLA‐DR, CD34 and CD45 and PE‐conjugated CD105, and rat anti‐human monoclonal antibodies: FITC‐conjugated CD44 (eBiosciences, San Diego, CA, USA) were used. As a control, isotype PE and FITC‐conjugated IgG were used. The cell suspension (1 × 10^6^ cells) was washed by PBS for twice and incubated with monoclonal antibodies at 4°C in the dark for 30 min. After washing with PBS, the samples were analysed by Cytomics FC 500 MPL cytometer (Beckman Coulter, Brea, CA, USA).

### Multilineage differentiation assays *in vitro*

Adipogenic, osteogenic, chondrogenic, neurogenic and cardiogenic differentiation was performed and identified as reported. Briefly, for differentiation assays, P3 MenSCs were suspended in growth medium and seeded at the density of 2 × 10^4^ cells/well in a 6‐well plate until confluence. Subsequently, the growth medium was changed to induction one (listed as follows), and the induction medium was replaced every 3 days except for neurogenic differentiation. Control cells were cultured in growth medium.

Adipogenic differentiation medium: growth medium + 1 μmol/l dexamethasone + 10 μg/ml recombinant human insulin + 200 μM indomethacin + 0.5 mM IBMX, for 14 days.

Osteogenic differentiation medium: growth medium + 0.1 μmol/l dexamethasone + 0.05 mmol/l ascorbic acid + 10 mM β‐glycerophosphate, for 21 days.

Chondrogenic differentiation medium: growth medium + 0.1 μmol/l dexamethasone + 0.2 mmol/l ascorbic acid + 1% insulin‐transferrin‐selenic acid + 10 ng/ml TGF‐β3, for 21 days.

Neurogenic differentiation medium: growth medium + 1 mM β‐mercaptoethanol, for 1 day; growth medium + 35 ng/ml all‐trans‐retinoic acid, for 3 days; growth medium + 5 ng/ml platelet‐derived growth factor + 10 ng/ml basic fibroblast growth factor + 14 μM fsk + 126 ng/ml GGF‐2, for 14 days.

Cardiogenic differentiation medium: growth medium + 20 μM 5‐azacytidine (Sigma‐Aldrich, St. Louis, MO, USA).

At the end of the induction period, cells were washed and fixed. Adipogenic differentiation was confirmed by oil red O staining; osteogenic differentiation was confirmed by alizarin red staining; chondrogenic differentiation was confirmed by alcian blue stainin neurogenic differentiation was confirmed by immunofluorescence assessment of GFAP (Abcam, USA); and cardiogenic differentiation was confirmed by immunofluorescence assessment of cTnT (Abcam, UK).

### Immunofluorescence

During a 5‐day culture, P0 MenSCs were fixed with 4% PFA for 20 min. and permeabilized with 0.05% Triton X‐100 for 10 min.; non‐specific binding sites were blocked using 5% goat serum for 30 min. Anti‐SOX2, anti‐SSEA4, anti‐c‐Myc and anti‐Nanog (Abcam) were separately added and incubated at 4°C overnight. Alexa Fluor 488‐conjugated goat antimouse or rabbit secondary antibodies (Life Technologies, USA) were incubated with the cells at 37°C for 1 hr. Cell nuclei were stained with DAPI. Finally, the cells were observed and imaged under fluorescence inverted microscope (Leica, Germany).

### Cell labelling and imaging

To track the transplanted cells after i.v. injection, P3 MenSCs were labelled by the fluorescent lipophilic tracer DiI (Molecular Probes, USA). For labelling, the cells were cultured in growth medium supplemented with DiI at a final concentration of 2.5 μg/ml before harvest. After incubation for 12 hrs, the cells were detached and centrifuged at 1000 r.p.m. for 5 min., washed twice with PBS and then adjusted to the concentration of 5 × 10^6^ cells/ml in PBS. For transplantation, 1 × 10^6^ DiI‐labelled cells (0.2 ml) were injected into BALB/c mice from tail vein (*n* = 3); the mice received 10 μg DiI in 0.2 ml PBS were taken as controls, and then, all the mice were killed after 72 hrs. The liver, lung, spleen and kidney were fixed in 4% formaldehyde solution overnight and then dehydrated in 18% sucrose solution overnight. Subsequently, the specimen was embedded in OCT compound (Sakura Finetek, USA), frozen in liquid nitrogen and stored at −80°C. Finally, the samples were adjacently sectioned with 20 μm thickness on the poly‐L‐lysine coated slides with a cryotome (Leica 1850) and imaged under a fluorescence microscope (Leica DFC425C).

### Immunogenicity

To examine the *in vivo* immune response to MenSCs, male BALB/c mice were randomly divided into three groups (*n* = 6): control group received 0.2 ml PBS by intraperitoneal injection, experiment group 1 received 1 × 10^6^ P3 MenSC in 0.2 ml PBS by intraperitoneal injection, and experiment group 2 received 1 × 10^6^ P3 MenSCs in 0.2 ml PBS by intravenous injection from tail vein. For examination, the blood samples were separately collected after 3 days and 7 days, and sent to Xinxiang Assegai Medical Laboratory Center (Xinxiang, China) within 8 hrs. Routine blood tests were performed by the ADVIA2120 haematology analyser (Siemens, Germany); the activity of associated enzymes (ALT, AST, CK and LDH) was determined by velocity method; the content of urea and creatinine (CR) was quantified by dehydrogenase and oxidase methods.

### Tumorigenicity

For determining tumorigenicity potential of MenSCs, nude mice were randomly divided into two groups (*n* = 5): MenSCs‐treated group (1 × 10^6^ P3 MenSCs in 0.2 ml PBS were injected subcutaneously into the right axilla of each nude mouse) and PC12 cells‐treated group (1 × 10^6^ PC12 cells in 0.2 ml PBS were injected subcutaneously into the right axilla of each nude mouse). The tumour formation was recorded at the time‐point of 1, 2, 3 and 12 weeks, respectively.

### Protein arrays

Angiogenesis and inflammation arrays (AAH‐CUST‐G1, RayBiotech, Norcross, GA, USA) were used according to the manufacturer's instructions to measure the expression levels of 11 angiogenesis‐associated biological factors and 11 cytokines in the conditional medium of MenSCs (*n* = 5). Adhesion molecule arrays (GSH‐CAM‐1) were used to measure the expression levels of 17 adhesion molecules on P3 MenSCs. Positive signals were captured on glass chips using a laser scanner (InnoScan 300 Microarray Scanner; Innopsys, Carbonne, France), and the observed fluorescence intensities were normalized to the intensities of the internal positive controls.

Preparation of the conditional medium of MenSCs: two million P3 MenSCs were seeded into 75 cm^2^ plastic cell culture flasks and cultured for 12 hrs, and then, the growth medium was changed to conditional medium (high‐glucose DMEM medium + 2% FBS + 100 U/ml penicillin + 100 mg/ml streptomycin). After being cultured for another 48 hrs, the conditional medium was collected and ten times concentrated by ultrafiltration.

### Statistical analysis

Results were presented as the mean ± S.D., and Student's paired two‐tailed test was used to determine statistical significance. *P* < 0.05 was considered to be statistically significant.

## Results

### Localization of MenSCs

**Figure 1:**
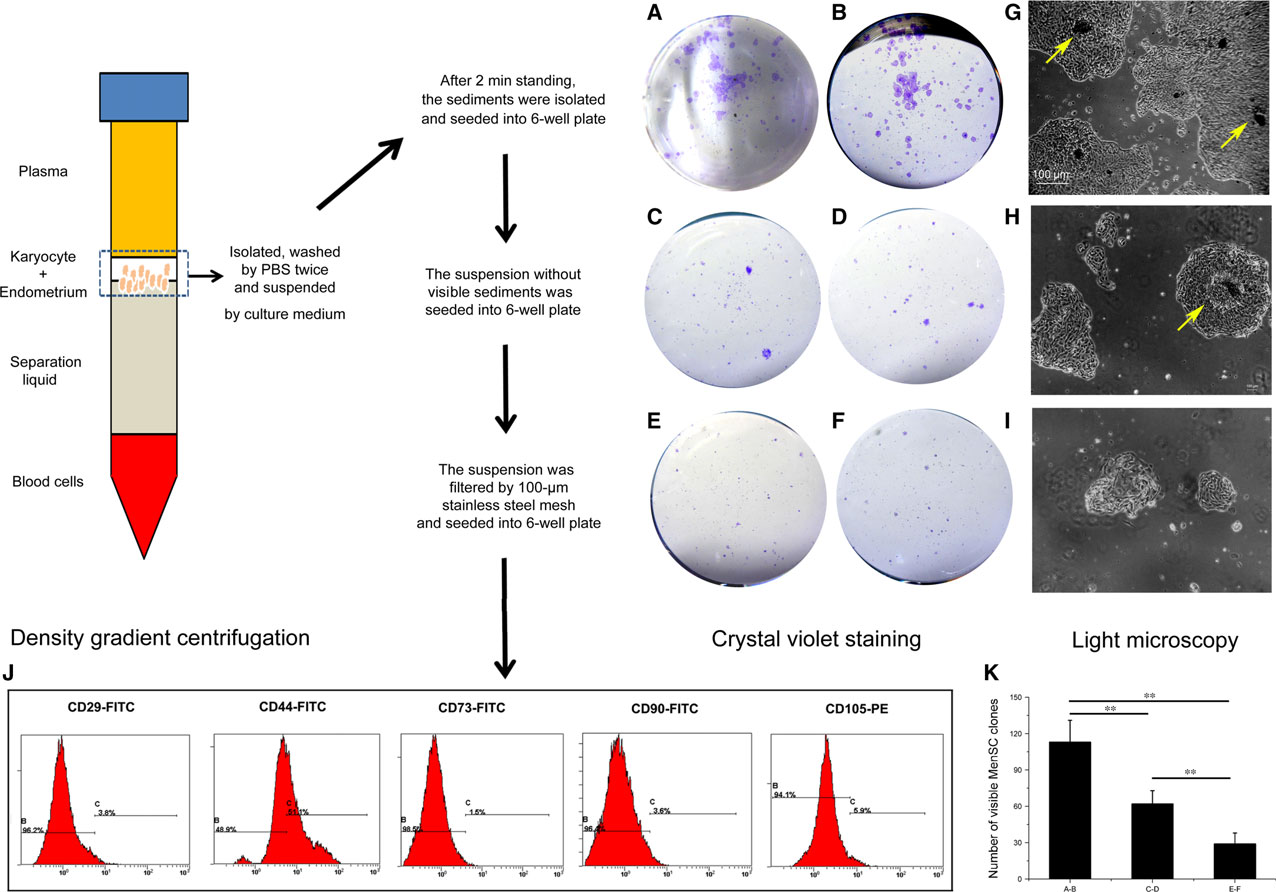
Localization of MenSCs. After density gradient centrifugation, the karyocytes and deciduous endometrium suspending in the buffy coat layer were collected, washed and resuspended in medium. The gross cell suspension was then standing still for 2 min. to allow the endometrium naturally sedimentating while karyocytes sustaining in the suspension. The suspension was then separated from the sediment: one portion was directly plated into 6‐well plate and the other portion was filtered with a 100‐μm filter before being plated similarly. The sediment was also plated into 6‐well plate. After 5‐day culture, the quantity and sizes of MenSC‐formed clones were determined by crystal violet staining. (**A** and **B**) the MenSC clones derived from the visible sediments after 2 min. standing; (**C** and **D**) the MenSC‐formed clones derived from the suspension without visible sediments before filtration; (**E** and **F**) the MenSC‐formed clones derived from the suspension after 100‐μm stainless steel mesh filtration. (**G–I**) morphologic images of MenSC‐formed clones photographed under microscope. The visible sediments (indicated by yellow arrows), which localized in the centre of MenSC‐formed clones, demonstrated that MenSCs originated from deciduous endometrium. (**J**) the karyocytes directly isolated from menstrual blood samples hardly expressed classical ASCs markers (<5%), which suggested that most MenSCs localized in deciduous endometrium. (**K**) quantification of MenSC‐formed clones in A–F (**P < 0.01). Scale bars: 100 μm in G for G–I.

Different from the conventional isolation of PBMC from peripheral blood, after density gradient centrifugation of menstrual blood, besides karyocytes, the flocculent membranes (deciduous endometrium) were also clearly observed in the buffy coat layer. For determining the localization of MenSCs, the procedures including natural sedimentation and stainless steel mesh filtration were used. As shown in Figure 1, the results demonstrated that the visible sediments (deciduous endometrium) could produce the most numbers of primary MenSC‐formed clones after conventional crystal violet staining (Fig. 1K). Meanwhile, we clearly observed that the tiny sediments (deciduous endometrium) localized in the centre of MenSC‐formed clones (indicated by yellow arrows in Fig. 1G and H). Also, the further FACS analysis demonstrated that the karyocytes suspended in buffy coat after being filtered through a 100‐μm stainless steel mesh were negative for classical ASC markers (<5%), such as CD29, CD73, CD90 and CD105 (Fig. 1J). All the above results confirmed that the MenSCs reside in endometrium which undergoes regular cycles of shedding and regeneration during menses.

### Identification of MenSCs

**Figure 2:**
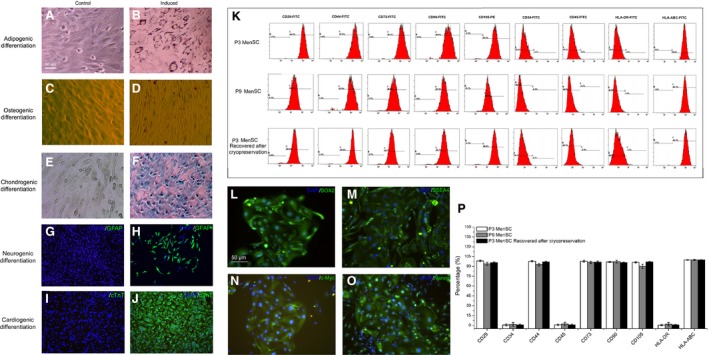
Identification of MenSCs. (**A–F**) the conventional adipogenic, osteogenic and chondrogenic differentiation was induced, and the results were demonstrated by positive oil red O, alizarin red and alcian blue staining, respectively (P3 MenSCs); (**G–J**) neurogenic and cardiogenic differentiation was performed, and the results were demonstrated by positive neurogenic marker (GFAP) and cardiogenic marker (cTnT) staining, respectively (P3 MenSCs). (**K**) the phenotype of MenSCs. To determine the immunophenotype of MenSCs, the cells with different passages (P3 and P9) were stained by corresponding conjugated antibodies and analysed by FACS. The P3 and P9 MenSCs positively expressed classical ASCs markers (CD29, CD44, CD73, CD90 and CD105) and HLA‐ABC; negatively expressed haematopoietic stem cells markers (CD34 and CD45) and HLA‐DR. (**P**) The quantification of flow cytometry results in Figure 2K. (**L–O**) MenSCs express typical ESC markers. After 5‐day culture, primary MenSCs were subjected to immunofluorescent staining of SOX2, c‐Myc, SSEA4 and Nanog. C: Scale bar: 50 μm in A for A–J and L–O.

During the first‐generation (Passage 0, P0) culture period, MenSCs formed colonies with radioactive or helix growth pattern outgrew the non‐adherent mononuclear cells, which were readily removable through refreshing the culture medium (Fig. 1G–I). After passaging, MenSCs displayed a typical spindle fibroblast‐like morphology for the whole culture period *in vitro* (Fig. 2A–C). Subsequently, the phenotype of these cells was evaluated with MenSCs from the third generation (Passage 3, P3) on and the exemplary FACS plots in Figure 2K show their positive expression of adult stem cell markers CD29, CD44, CD73, CD90, CD105 and HLA‐ABC. Importantly, MenSCs expressed neither haematopoietic cell marker CD45 nor haematopoietic stem cell marker CD34, nor type II antigen presentation molecule HLA‐DR. Thus, the MenSCs we obtained display ASC phenotype and this phenotype remains during cryopreservation and further division (Fig. 2P). Several researchers have observed that MenSCs are closer to embryonic stem cells than other types of ASCs, as they express OCT‐4, SOX2 and SSEA‐4 11, 12, 13. Our data also confirmed this acclaim, as MenSCs are positive for SOX2, SSEA4, c‐MyC and Nanog (Fig. 2L–O). To further prove the stem cell identity of MenSCs, we tested their pluripotency by culturing them with various types of differentiation medium. As shown in Figure 2A–J, MenSCs underwent adipogenic, osteogenic, chondrogenic, neurogenic and cardiogenic differentiation, respectively, demonstrating their stem cell nature.

### The number of passages negatively influences the proliferation capacity of MenSCs

**Figure 3:**
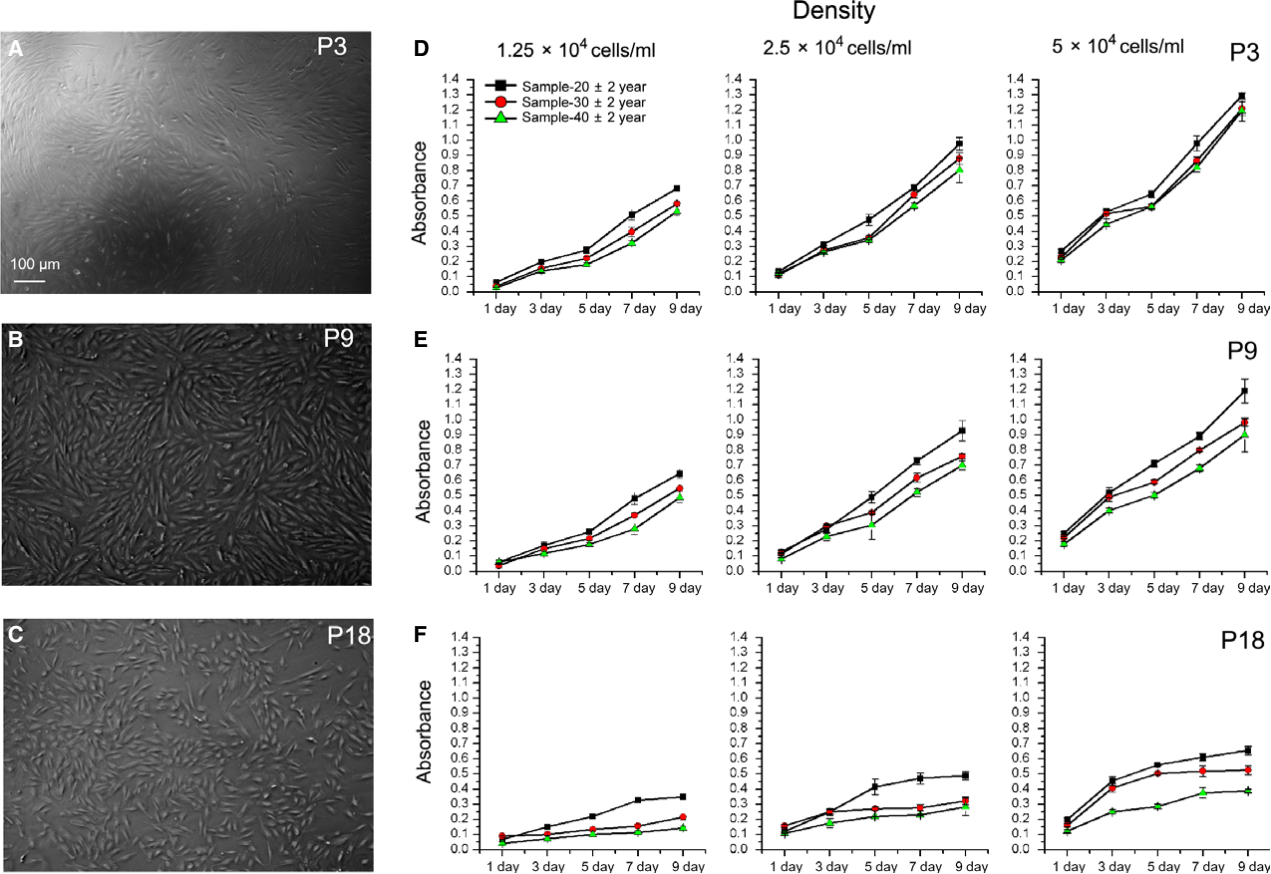
Influence of donor age and passage times to proliferation capacity of MenSCs. (**A–C**) The representative morphology of P3, P9 and P18 MenSCs; (**D–F**) P3, P9 and P18 MenSCs derived from 20 ± 2‐, 30 ± 2‐ and 40 ± 2‐year‐old donors (*n* = 6) were seeded into 96‐well plate with indicated cell density, and the proliferation capacity during the 9‐day culture was determined by MTT assay. Scale bars: 100 μm in A for A‐C.

Menstrual blood was collected from donors with different age: 20 ± 2‐, 30 ± 2‐ and 40 ± 2‐year old, respectively (*n* = 6). MenSCs were isolated, and their proliferation capacities were evaluated when seeded at various densities with cells that were maintained at various passages (P3, P9 and P18). During early passage (P3), the proliferation rates of MenSCs from differently aged donors were only slightly different, in an age‐coupled fashion: the older the donor, the slower the proliferation, especially at low density (Fig. 3D). Remarkably, following further division (P9 and P18), this difference becomes obvious. Of note, the MenSCs of P18 proliferated much slower than cells of P9 and P3 (Fig. 3E and F). Therefore, the age of the donor and times of the passages negatively correlate with the proliferation capacity of MenSCs.

### A three‐day storage of menstrual blood prior to stem cell isolation is permitted

**Figure 4:**
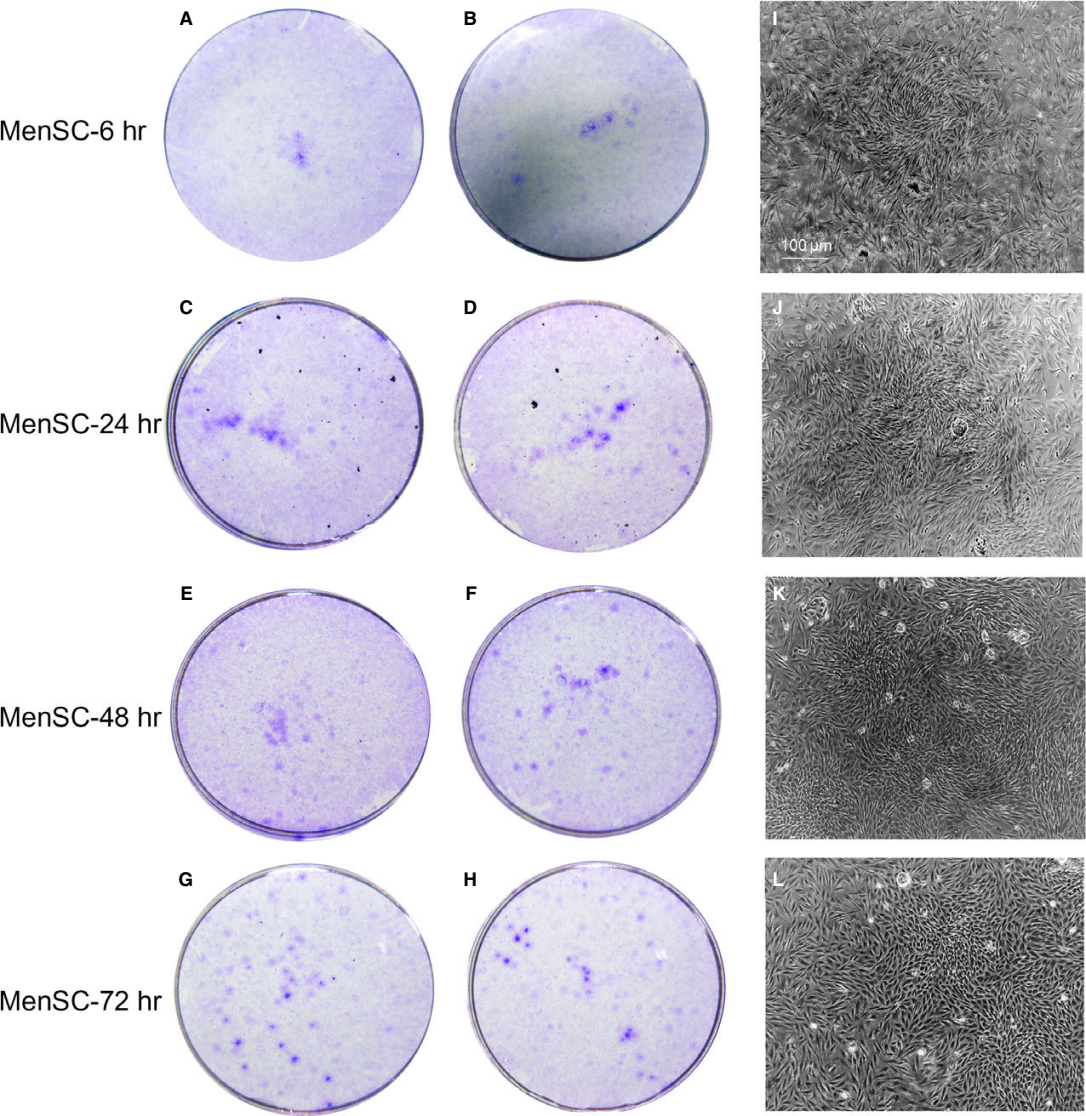
Impact of the storage duration of menstrual blood sample prior to isolation on the viability of MenSCs. The menstrual blood samples were immediately divided into four equal portions after collection and stored at 4°C. MenSCs were then isolated 6 hrs (**A** and **B**), 24 hrs (**C** and **D**), 48 hrs (**E** and **F**) and 72 hrs (**G** and **H**) later. After 5‐day culture, the quantity and size of MenSC‐formed clones were determined by crystal violet staining (A–H). In addition, morphology of MenSC‐formed clones was imaged under microscope (**I–L** for 6 hrs, 24 hrs, 48 hrs and 72 hrs, respectively). Scale bars: 100 μm in I for I‐L.

To evaluate the effects of the storage duration of menstrual blood prior to the isolation of MenSCs on their viability, the collected menstrual blood samples were equally divided into four portions and stored at 4°C. MenSCs were then isolated 6, 24, 48 and 72 hrs later, respectively. As shown in Figure 4, no major differences with regard to the morphology of MenSCs were observed. Furthermore, the classical characteristics of ASCs were routinely examined, such as proliferation capacities, phenotypical marker expression, and differentiation potential were tested and showed no significant difference among the MenSCs isolated after these different duration of storage. Thus, menstrual blood samples can be kept at 4°C for at least 3 days before being processed further, giving scientists a comfortable time frame to schedule experiments.

### MenSCs impose little immunogenicity and tumorigenicity after transfer

Stem cell‐based therapy requires host tolerance to the stem cells transferred. To evaluate the immunogenicity of MenSCs, we transferred MenSCs *via* i.v or i.p. and measured the indicators of pathologic status of liver, heart and kidney (ALT, AST, CK, LDH, urea and CR) during MenSCs transfer. No major alteration was observed. Thus, MenSCs transfer has little adverse effects on the immune system of recipients (Table 1).

**Table 1:** Activity of pathology‐related enzymes and content of urea and CR at 3 and 7 days after MenSCs transplantation *via* i.v and i.p

	Control group (*n* = 6)	MenSCs‐treated groups (*n* = 6)			
		i.v for 3 days	i.v for 7 days	i.p for 3 days	i.p for 7 days
ALT(U/l)	45 ± 13.12	59 ± 17.21	42 ± 11.36	61 ± 12.58	33 ± 13.21
AST(U/l)	87 ± 16.43	105 ± 21.33	94 ± 19.87	93 ± 16.31	83 ± 20.41
CK(U/l)	472 ± 272.55	563 ± 264.31	395 ± 186.57	548 ± 201.39	345 ± 256.48
LDH(U/l)	524 ± 167.82	470 ± 210.43	468 ± 195.28	483 ± 201.78	577 ± 186.49
Urea (mmol/l)	9.9 ± 2.23	8.6 ± 1.99	9.2 ± 2.01	10.3 ± 1.85	7.9 ± 2.72
CR(μmol/l)	10 ± 2.21	10 ± 1.89	7 ± 2.77	9 ± 1.91	8 ± 2.62

ALT, alanine transaminase; AST, aspartic acid transaminase; CK, creatine kinase; LDH, lactate dehydrogenase; CR, creatinine.

**Figure 5:**
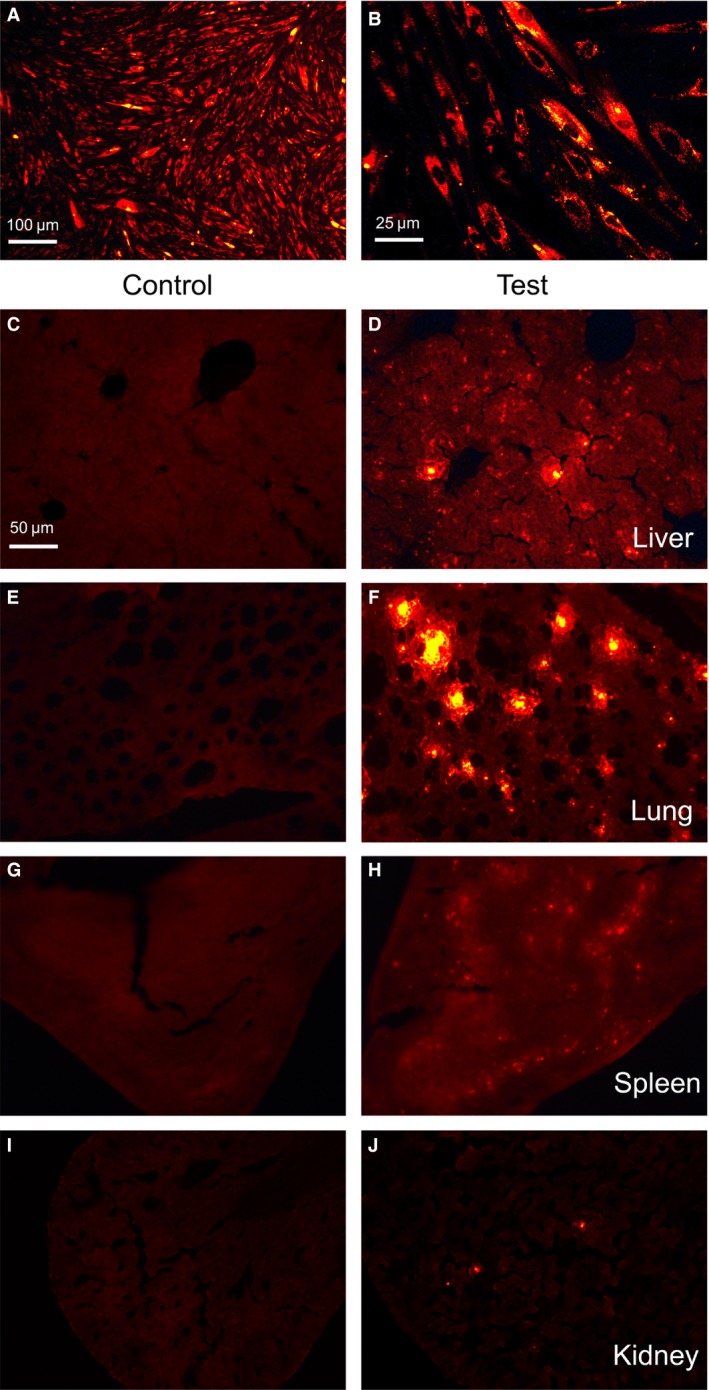
Organic distribution of MenSCs after being injected into mouse through tail vein. P3 MenSCs were labelled with DiI (**A** and **B**) before injection. In 72 hrs after the injection, MenSCs were found in lung and liver (**D** and **F**) and to a small extent, in spleen (**H**), but almost not in kidney (**J**), (**C**,** E**,** G** and **I**) served as controls in relative organs, respectively. Scale bars: 100 μm in A; 25 μm in B; 50 μm in C for C–J.

A few studies documented that MenSCs have much weaker tumorigenicity *in vitro* and *in vivo,* compared to embryonic stem cells 12. We also attempted to address this point using immunodeficient strain: nude mice. As shown in Figure 5, the tumour became visible when mice received pheochromocytoma cell line PC12 cells only 1 week after inoculation, continuously grew thereafter and killed the recipients around 12 weeks later. In comparison, nude mice that received MenSCs remain tumour‐free till the end of the observation frame (week 12).

### Organic distribution of MenSCs after adoptive transfer

**Figure 6:**
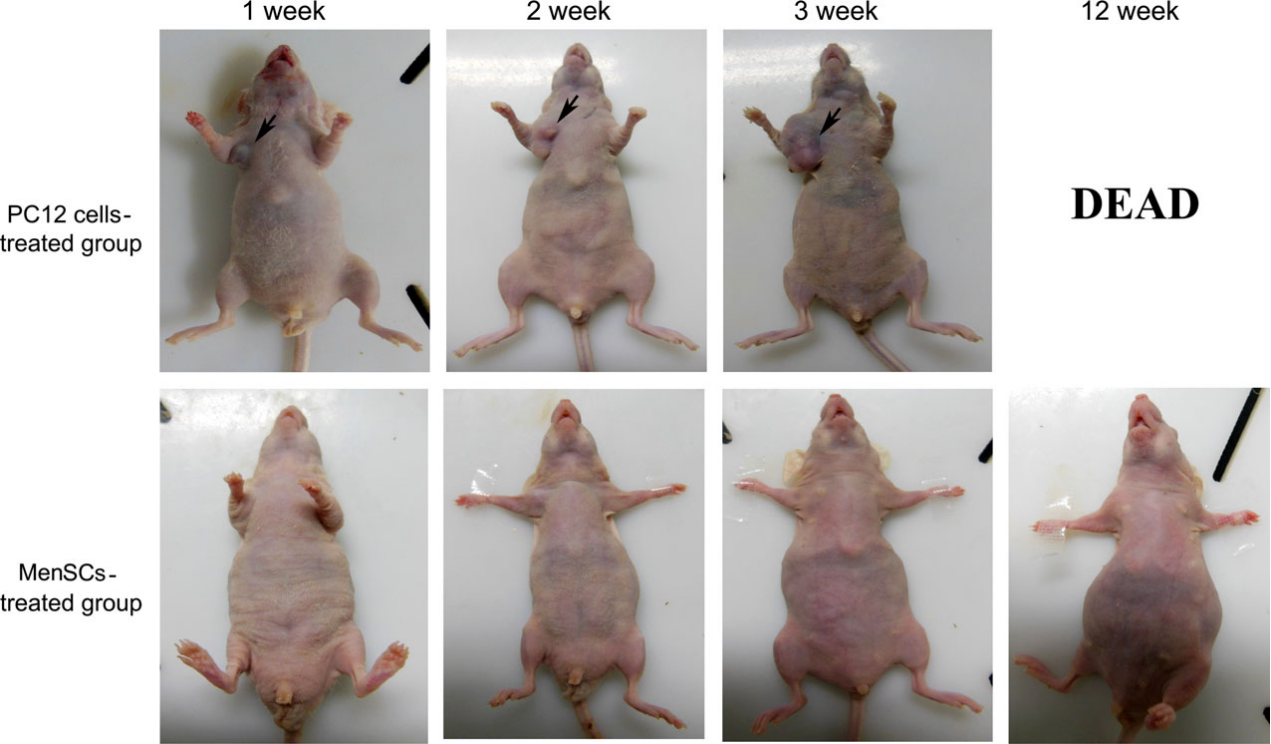
MenSCs have little tumorigenicity. 1 × 10^6^ PC12 cells or P3 MenSCs were injected subcutaneously into the right axilla of nude mice (*n* = 5). The nude mice which received PC12 cells started to generate tumours 1 week thereafter and the tumours continuously grew during the following weeks and eventually killed the recipients at week 12. In contrast, the nude mice which received MenSCs remained healthy during the whole observation phase.

*In vivo* localization of stem cells after transfer is important for interpreting the functions of these cells. We seek to define the distribution of MenSCs after being transferred into mice. To this end, we first labelled MenSCs with DiI and transferred them *via* the tail vein into mice; 72 hrs later, we analysed various organs and observed that MenSCs mainly be detained in the lung and liver, some in spleen and only a few appeared in the kidney (Fig. 6).

### Paracrine production of biological factors in MenSCs

**Figure 7:**
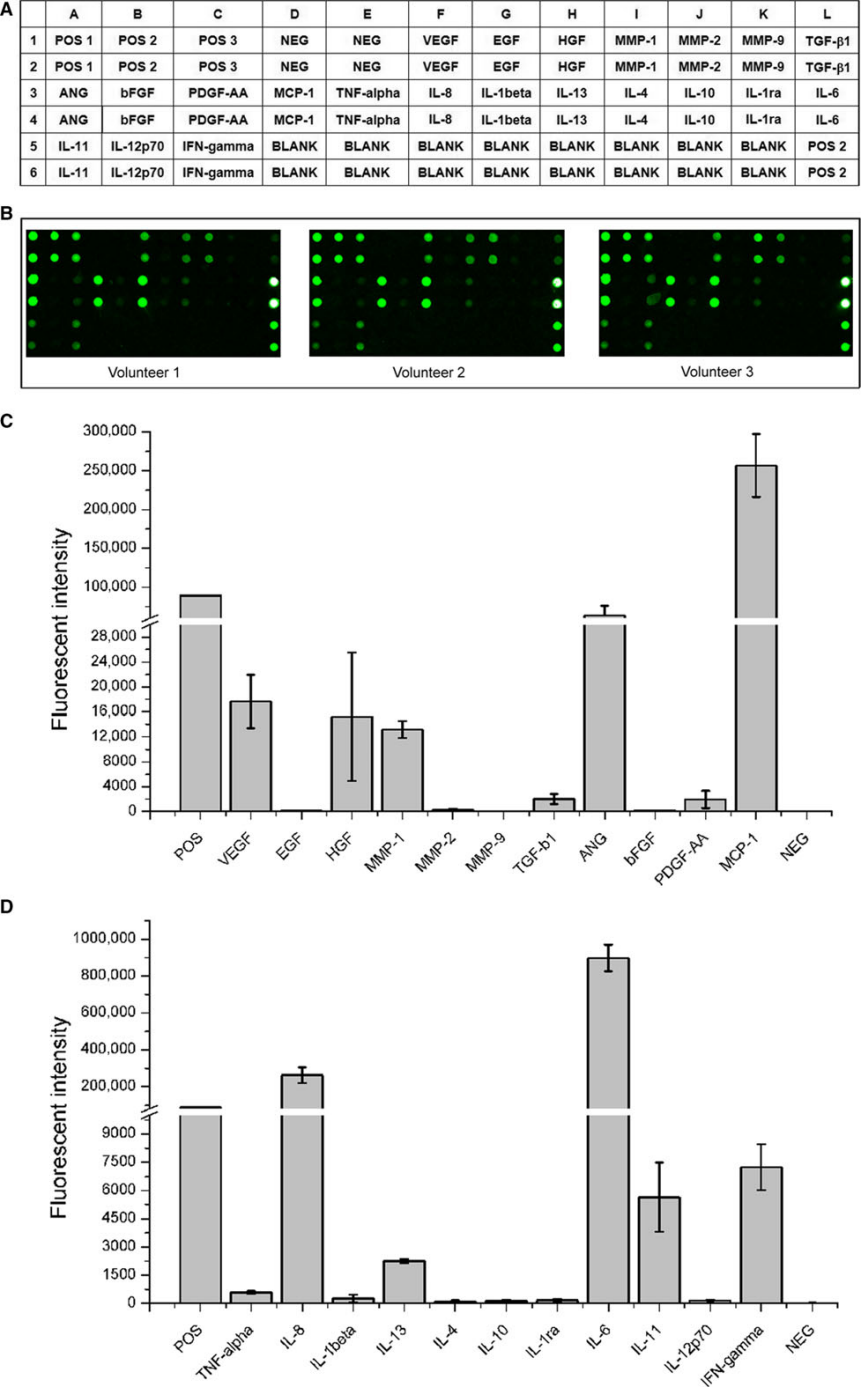
Paracrine production of biological factors in the conditional medium of MenSCs. (**A**) the map of factors examined. (**B**) three representative array images are shown (*n* = 5). (**C** and **D**) the fluorescence intensities of the indicated biological factors.

To identify the angiogenic and immunomodulatory factors secreted in the conditional medium of MenSCs, a protein array was used to examine the levels of associated biological factors (Fig. 7A and B). Besides typical angiogenic factors, including vascular endothelial growth factor (VEGF), hepatocyte growth factor (HGF) and angiogenin (ANG), matrix metalloproteinases 1 (MMP‐1) and monocyte chemoattractant protein (MCP‐1) also were expressed at higher levels in the conditional medium of MenSCs (Fig. 7C). In addition, compared to anti‐inflammatory cytokines (such as IL4, IL10 and IL13), the pro‐inflammatory cytokines (such as IL‐6, IL‐8 and IFN‐gamma) were expressed at higher levels in the conditional medium of MenSCs (Fig. 7D).

### Expression of adhesion molecules on MenSCs

**Figure 8:**
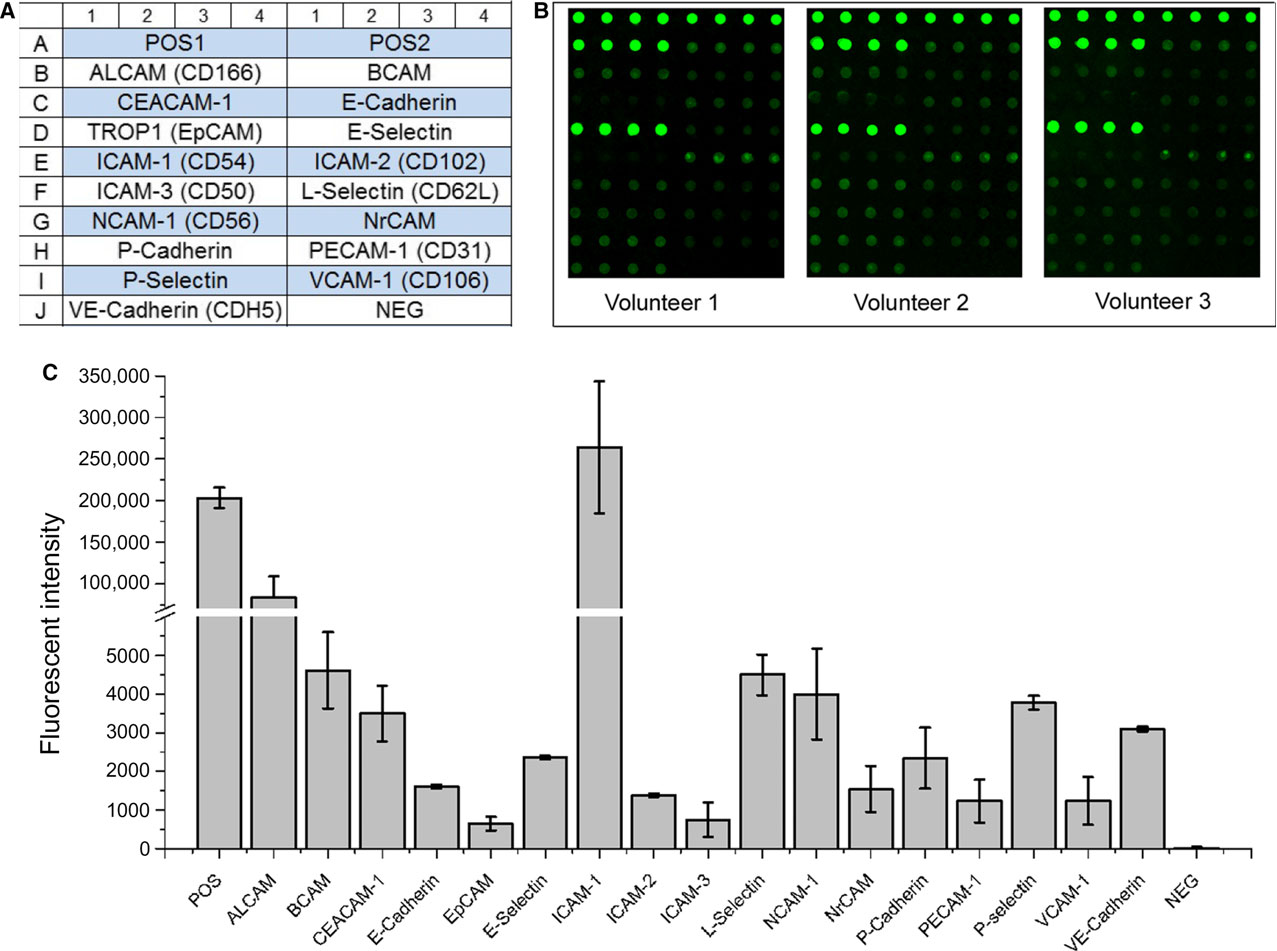
Expression of adhesion molecules on MenSCs. (**A**) the map of adhesion molecules examined. (**B**) three representative array images are shown (*n* = 5). (**C**) the fluorescence intensities of the indicated biological factors.

The adhesion molecules expressed on MenSCs were also determined by protein array. As shown in Figure 8C, among the 17 adhesion molecules that were evaluated in this array, in addition to highly expressed activated leucocyte cell adhesion molecule (ALCAM) and intercellular cell adhesion molecule‐1 (ICAM‐1), MenSCs still moderately expressed basal cell adhesion molecule (BCAM), carcinoembryonic antigen‐related cell adhesion molecule 1 (CEACAM‐1), L‐selectin, neural cell adhesion molecule (NCAM), P‐selectin and vascular endothelial cadherin (VE‐cadherin).

## Discussion

ASCs‐based therapy is a rapidly evolving field for diseases without effective treatments, and during the past decade, strong evidence has been accumulated including pre‐clinical and clinical trials ranging from proof‐of‐concept to phase II trials 19, 20, 21. As an alternative source of human stem cells, the newly identified MenSCs exhibit their potential for ASC‐based therapy. Due to the natural high richness and non‐invasive sample collection, MenSCs display tempting features as replacement of bone marrow‐derived stem cells 22, 23, 24.

So far, the successful isolation of MenSCs from menstrual blood samples has been demonstrated, but the detailed processes are always simplified 9, 11, 25. Here, we directly proved that MenSCs reside in deciduous endometrium, and suggested that MenSCs should have similar nature with endometrial stem cells (EnSCs). The subsequent results demonstrated that MenSCs could be successfully isolated from the samples stored at 4°C even for 72 hrs, which not only provide support for the MenSCs isolation, but also guarantee the privacy of donors and menstrual blood samples transportation by express. Generally, the MenSCs used in most of the published studies are isolated from volunteer donors with different age, and it is reasonable to assume that the characteristics (proliferative capacity, stem potential and differentiation potential) of MenSCs derived from differently aged donors are distinct, and the viability of MenSCs derived from younger donors is likely more promising than the older ones 26. As documented in this study, our results suggested that the MenSCs isolated from younger donors may become a practical solution for the allogeneic MenSC‐based therapies. Furthermore, MenSCs at earlier passages proliferate faster than at later passages, and the periodic collection of menstrual blood ensures a high therapeutic dose and repetitive treatment of MenSCs at their early passages, further speaking for the prestige of MenSCs as a source of stem cell therapy.

Ideally, autologous transplantation of MenSCs is the best way to treat the female patients who need, but the real clinical situation is often hard to deal with 27, 28, 29. This is because most of the patients who need stem cells‐based therapy are too old (menopause) to collect menstrual blood, and the evidence regarding the availability of MenSCs isolated from endometrial biopsy in post‐menopausal women has not been reported; moreover, the patients with acute disease may be not able to endure the whole process of isolation and proliferation of enough MenSCs. Consequently, in view of intrinsic low immunogenicity of MenSCs, allogenic transplantation may be the most practical and useful way to treat the patients who need. However, the current clinical trials seem to be moving ahead of the pre‐clinical investigations and without adequate evaluation of its impact on large animal models, which are needed to guide the extensive application of MenSCs in clinic 27, 28, 29, 30, 31.

Therefore, it is crucial to evaluate the safety of MenSCs transplantation, which is another research focus in our study. As also demonstrated by other research groups, no evidence of tumour growth was found in inoculation site until 12 weeks, and in contrast, the tumours were visible in positive control group (PC12 cells‐treated group); moreover, the further biochemical indicators tests also demonstrated no adverse effect on the function of key organs after MenSCs transplantation. These findings not only provided support for that MenSCs which are immune‐privileged, but also suggested the safety of allogeneic MenSCs transplantation in further clinical application 22, 23. In addition, MenSCs' distribution *in vivo* also provides support for MenSCs‐based therapies for the diseases in lung or liver, where MenSCs would preferentially and mainly reside.

It is well known that paracrine functions performed by ASCs play therapeutic roles in ASC‐based therapy. Also, the transplantation of synthetic cell‐mimicking microparticle (CMMP), in which the secreted proteins isolated from cardiac stem cells or bone marrow‐derived mesenchymal stem cells were packaged by associated stem cell membrane, could improve cardiomyocyte functions without immune activation 32, 33. So under the premise of safety of MenSCs transplantation, the paracrine production of biological factors secreted by MenSCs and cellular adhesion molecules expressed on MenSCs were examined by protein array. In accordance with previous report 34, we found that MenSCs cultured *in vitro* secreted higher levels of angiogenic factors (VEGF, HGF, ANG and MMP‐1); meanwhile, the pro‐inflammatory cytokines (L‐6, IL‐8 and IFN‐gamma) were expressed at higher levels than anti‐inflammatory cytokines (IL‐4, IL‐10 and IL‐13), which could recruit endothelial cells and associated leucocytes to sites of injured tissues. Although the above results confirmed the angiogenesis‐promoting and immunomodulatory potential of MenSCs through paracrine effect *in vitro*, the therapeutic effect *in vivo* still need to be testified in animal models because of the huge difference in microenvironment.

Furthermore, adhesion molecules analysis demonstrated the higher expression of ALCAM and ICAM‐1 on MenSCs. ALCAM plays a role in the binding of T and B cells to activated leucocytes, as well as homophilic adhesion (ALCAM‐ALCAM) was shown to play important roles in tight cell‐to‐cell interaction and regulation of stem cell differentiation. Simultaneously, ICAM‐1 also plays a role in the binding of leucocytes through binding to LFA‐1 (lymphocyte function‐associated antigen 1), and recently, it is reported that low‐level expression of ICAM‐1 on ASCs has a critical impact on ASC‐mediated immunosuppression 35, 36. Therefore, the high expression of adhesion molecules mentioned above provided support for the immunomodulatory effects of MenSCs through directly binding to leucocytes.

In conclusion, our findings not only confirmed the biological characteristics of the MenSCs, such as localization, typical ASC's morphology, high proliferative capacity, classical ASC's surface markers and multilineage differentiation potential, but also suggested the safety of MenSCs‐based therapies. Furthermore, protein arrays suggested that MenSCs may have potentially therapeutic effects on diseases through paracrine effect and immunomodulation. These complete characterizations of MenSCs have improved our understanding of their biological features and provide alternative, readily available therapeutic approaches for human diseases. Then, the further pre‐clinical studies regarding the standardization of the MenSCs isolation and proliferation, storage *in vitro*, practical delivery methods, the dosing of the cells and adjuvant strategies will have to be performed before MenSCs are used routinely in the wider field of regenerative medicine.

## Author contributions

J. L. and Y. L. were the principal investigators and take primary responsibility for the paper. J. L., Y. L. and P. S. conceived and designed the experiments. Y. L., R. N., F. Y., Y. S., S. L. and Y. Y. performed the experiments. J. L., Y. L. and P. S. analysed the data and wrote the paper and prepared figures. All authors reviewed this manuscript.

## Conflict of interests

The authors declare that there is no conflict of interests regarding the publication of this paper.
